# Experiential education enhancing paramedic perspective and interpersonal communication with older patients: a controlled study

**DOI:** 10.1186/s12909-018-1341-9

**Published:** 2018-10-20

**Authors:** Linda J Ross, Paul A Jennings, Cameron McR Gosling, Brett Williams

**Affiliations:** 1 Department of Community Emergency Health & Paramedic Practice, Monash University, PO Box 527, McMahons Road, Frankston, VIC 3199 Australia; 2 Ambulance Victoria, 375 Manningham Rd, Doncaster, VIC 3108 Australia

**Keywords:** Allied health personnel, Emergency medical technicians, Paramedic, Psychosocial support systems, Aged, Older patients, Older people, Communication, Controlled before-after studies

## Abstract

**Background:**

Paramedics are required to provide care to an aging population with multidimensional and complex issues. As such educators need to prepare undergraduate paramedics to recognise, assess and manage a broad range of psychosocial care and support issues beyond somatic conditions. Experiential educational interventions with older people provide realistic and contextualised experience which can improve the provision of holistic patient focused care.

**Methods:**

This was a single institution controlled before-after study with parallel groups, conducted in Australia in 2017. It was designed to compare the effectiveness of an educational program related to older people (intervention), verses no intervention (control) on paramedic student attitudes, knowledge and behavior with older patients.

**Results:**

A total of 124 second year paramedic students were included in this study; 60 in the intervention and 64 in the control group. Their demographics and Time 1 baseline results were homogeneous. Both groups showed improvement in communication skills with real older patients (*p* < 0.001, η^2^ = 0.41) and (*p* < 0.001, η^2^ = 0.35). The intervention group showed greater improvements in the ‘understands the patient’s perspective’ element for both the self-assessment (*p* < 0.001) and the clinician assessment (*p* = 0.01). Multiple linear regression Model 1 found gender (β = − 0.25; *p* = 0.01) was the best predictor of clinician-assessed communication, with females having higher scores. Knowledge and attitudes remained relatively unchanged for both groups.

**Conclusions:**

As the first study to observe, measure and report on the interpersonal communication skills of paramedic student’s with ‘real’ older patients we can report that these skills were from fair to good at baseline and improved from good to very good post the intervention. Overall improvement was notably better in the ‘understanding the patients perspective element’ for the intervention group who had conducted one-one visits with an older person.

## Background

Undergraduate paramedic education aims to equip students with the knowledge, behaviours and skills required to provide competent and compassionate patient care. Current paramedic programs in Australia are gradually progressing toward teaching a biopsychosocial approach to patient care which recognises the importance of treating patients holistically [1–3]. This has never been more important than with older patients (≥65) who are more likely to suffer from multiple conditions across the biopsychosocial spectrum [4, 5]. It is imperative therefore that paramedic graduates have awareness and understanding of issues that impact older people beyond physical problems in order to develop appropriate recognition, assessment and management skills.

It has been suggested that younger adults lack life experience, awareness of diverse communities in which they will work, and interpersonal skills [6, 7]. The median age of paramedics students in Victoria, Australia was 21 years in 2015 [8]. In addition they are drawn to a career often misrepresented in the media as action packed with dramatic rescues, life and death events and emergency driving [9]. For example, a single institution study of 168 paramedic students found the top three motivating factors for wanting to become a paramedic were ‘wanting to help people’, ‘saving lives’ and ‘an exciting career’ [10]. They are thus highly motivated when it comes to learning and practising clinical concepts and advanced life support skills, with these aspects prioritised and seen to be more important than constructs such as interpersonal communication [11].

Exceptional interpersonal communication skills are essential as they allow for the development of clinician-patient rapport, which facilitates the sharing of information, compliance with treatment and overall patient satisfaction [12–14]. While educators endeavour to teach the value of interpersonal communication and the associated skills, the links between this and patient outcomes is poorly established upon graduation [6, 7, 15].

An individual’s behaviour toward others can be influenced by experience, knowledge, awareness, prejudice, attitudes, and confidence [16]. The interpersonal communication skills of paramedics and other health care professionals are no exception. For example, Ajzen’s Theory of Planned Behaviour (TPB) asserts that attitudes are formed through knowledge and experience, and that there is a causal relationship between attitudes, intentions, and behaviour [16]. The ability of paramedic students to communicate compassionately and effectively with older patients is therefore influenced by their past experience, knowledge and ultimately attitudes toward them.

Teaching interpersonal communication skills is challenging, with tradition didactic methods having limited success [17]. If, as the TPB attests, the key to changing behaviour is through improving attitudes, it is necessary to implement educational strategies that target knowledge and experience [18]. Attitudes toward older adults improve best by enhancing awareness, knowledge and understanding [19]. A systematic review of educational interventions designed to improve health care student attitudes toward older adults found that interventions incorporating interactions with independently living real patients had the most positive impact [20]. This is supported by Kolb’s Experiential Learning Theory (ELT) whereby students learn and develop attitudes best when they are in touch with the realities and gain contextualised experience [21]. Such experiential interventions should, in theory and practice, translate to better attitudes and behaviour.

Previous research has highlighted paramedic students have varied experience, limited knowledge and slightly positive attitudes toward older patients [8]. It is unknown however, if and how knowledge and attitudes translate to behaviour. This current study is one of few to report on observed behaviour of health care students toward older adults, and the first to have observed and analysed paramedicine. The aim of this study was to determine the effects of an educational intervention with older people on student paramedic’s knowledge, attitudes and behaviour toward older patients.

## Methods

### Study design

This was a single institution controlled, before-and-after tri with parallel groups conducted in Australia between Feb – May 2017 (Semester 1). It was designed to compare the effectiveness of an educational program related to older people (intervention), verses no intervention (control) on paramedic student attitudes, knowledge and behavior with older patients. As the educational intervention was embedded within the undergraduate paramedic curriculum it was repeated between July – October 2017 (Semester 2) to ensure the control group received the same program. The study was approved by the Monash University Human Research Ethics Committee (MUHREC - 2016-1370).

### Participants and setting

The participants were 2nd year Bachelor of Emergency Health and Paramedic Practice students from Monash University in Melbourne, Australia. All students had to be concurrently enrolled in two units of study; EPP2011 ‘Clinical concepts of paramedic practice 2’ and HSC2200 ‘Health and the human lifespan’ to be eligible to participate in the study. The interventions were embedded components of these units therefore active recruitment was not required. Prior to the Time 1 data collection students were given an explanatory statement about the study and completed and signed a consent to participate form.

#### Sample size

A power calculation using G*Power (Version 3.1.9.2, F.Faul, Germany) determined 64 participants per group would be required to detect a difference between groups, with a two-tailed α of 0.05, an effect size (d) of 0.5 and a (1-β) of 0.80.

### Procedures

#### Group allocation

Students were allocated based on their tutorial group for HSC2200. Three tutorial groups were assigned to the intervention arm and three to the control arm. Students were allocated by the university timetabling software to a tutorial group based on their preferences and other timetabled classes.

#### Blinding

The research team were blinded to the group allocation throughout the process. The group allocation was done by the university timetabling system and the intervention was delivered and administered by teaching staff not involved in the study. Students were aware of their allocation once they began the intervention and were asked not to share intervention details with those in the other group.

#### Intervention

##### Part 1. Geriatric respect, awareness, care and compassion (GRACC) workshop

This two-hour workshop included a small group activity to discuss and answer 10-multiple choice questions on demographic and biopsychosocial factors pertinent to older people. It also included viewing footage of older people telling their stories, followed by discussion about physical and emotional needs and the impact of listening and ‘being heard’. It concluded with some small group role playing exercises simulating paramedics attending older patients. This workshop was designed by the research team to equip the students with greater knowledge and awareness of older people, and some tools to communicate effectively with them prior to part 2 of the intervention.

##### Part 2. Geriatric home visits

Following the GRACC workshop participants were asked to seek out an older adult from the community for 4 × 1 hr visits. The older adult needed to be able to communicate, and not be related to the participant. Participants were instructed to keep these visits relatively unstructured, while aiming to get to know the person, gain awareness of what makes them ‘tick’, what is important to them, and what communication strategies work best with them. A series of potential questions or conversation starters were made available.

#### Control

The control group participated in a similar workshop about paediatric patients and conducted home visits with children.

### Instrumentation

Three instruments were used to collect data from the participants at Time 1 (pre-intervention Feb 2017), and Time 2 (post-intervention May 2017).

1. Aging Semantic Differential (ASD) is a widely used validated instrument to assess stereotypical attitudes towards the older people [22–24].

2. Facts on Aging Quiz 2 (FAQ2) is a brief, reliable, easily administered test of factual knowledge on aging [25]. The Australian version of the Facts on Aging Quiz 2 (FAQ2) was used in this study [26].

3. Kalamazoo Communication Skills Assessment (KCSA) is a modified version of the original Kalamazoo Essential Elements Communication Checklist [27]. It is a communication skills assessment tool with good internal consistency [28]. Originally designed for physicians it was modified from 9 to 6 communication elements pertinent to paramedic-patient communication (i.e. 3 elements relevant only to physician practice were removed).

### Older people recruitment and training

Five independently living older people with a mean age of 73 were recruited from the community via email and word of mouth. Prior to the Time 1 data collection the older people underwent a one-hour training session covering what to expect, and what was required of them. For the encounters they were given a brief script with the reason for calling the paramedics. Aside from the reason for the paramedic call all other answers regarding past medical history, allergies etc. were their own. This assisted the older people to be themselves without the need to act, remember detailed information, or take on an unfamiliar role. They were blinded to the group allocations.

### Clinical rater recruitment and training

Clinical raters were recruited from paramedic educators teaching into the Bachelor of Emergency Health and Paramedic Practice. Three different raters with an average of 10 years clinical experience were used. They were given a summary of the project and instructions to rate the encounter based on what they would expect of an ‘average’ qualified paramedic. They were also blinded to group allocation.

### Piloting

Utilising a staff member in place of a real older patient, three staff and three post-graduate students completed the patient-centred interview and surveys. The process was timed and feedback sought. It was determined that in addition to the 10 min patient-centred interview the surveys would take between 10 and 15 min. Feedback lead to the FAQ2 questions being reduced from 25 to 20 due to relevance to the Australian paramedic context.

### Outcomes – Primary and secondary

The primary outcome for this study was paramedic student behaviour toward older adults manifest in interpersonal communication. This was assessed by the KCSA which was completed by 3 raters; the student, the patient and a clinician following a 10 min patient-centred interview with an older adult at both Time 1 and Time 2.

Secondary outcomes included attitudes towards older adults (assessed via the ASD) and knowledge about older adults (assessed via the FAQ2). Both these self-report measures were completed by the participant prior to the interview with the older patient at both Time 1 and 2.

Students were randomly selected during their EPP2011 practical class and asked to complete demographic details, the ASD and FAQ2. They were then dispatched to a fictitious case involving an older person and asked to conduct a patient-centred interview within 10 min. After this the student participant and patient both completed the KCSA. These encounters were videoed allowing a clinician to view and complete a KCSA at a later time.

### Data analysis

Data was stored and analysed using the Statistical Package for the Social Sciences (SPSS Version 23, IBM Corp, Armonk, NY). Mean and standard deviation or median and interquartile ranges were used to report data as appropriate. Independent sample t-tests were used to compare the intervention and control groups. Paired sample t-tests were used to compare the results of both groups’ pre and post the intervention. KCSA scores for all 6 elements were treated individually, and totaled out of 30 as the outcome variable to determine factors that predict the total score. Linear regression models were used to analyse the relationship between independent variables and total KCSA scores. Internal consistency of each scale was measured with Cronbach α. All tests were 2-tailed and results were considered statistically significant at *p* < 0.05. Eta squared (η2) = 0.01, 0.06, 0.14 represented small, medium and large effect size respectively.

## Results

### Participant demographics

**Fig. 1:**
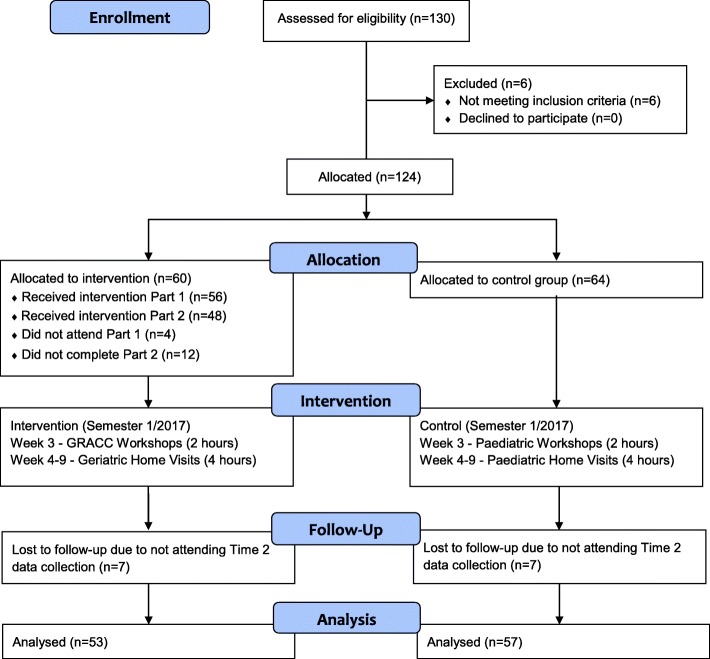
Flow diagram of progress through the study

Of the 130 second year paramedic students, 124 were eligible to participate in this study. Their flow through the allocation and intervention is shown in Fig. 1 [29]. The demographics of both groups were homogenous. Of the intervention group the median (interquartile range) age of students was 20 (19–24) years, 62% (37/60) female. Students in the intervention and control groups encountered a similar number of geriatric patients on placements, (mean ± SD: 6.62 ± 2.67 and 6.75 ± 2.59) respectively. Full demographic details are reported in Table 1.

**Table 1:** Student Demographics & Participation (*N* = 124)

	Intervention (*n* = 60)	Control (*n* = 64)
AGE		
Md (IQR)	20.0 (19–24)	19.5 (18–30)
Min - Max	18–34	18–30
GENDER		
Male	23 (38%)	29 (45%)
Female	37 (62%)	35 (55%)
GERIATRIC WORKSHOP		
Yes	56 (93%)	0 (0%)
No	4 (7%)	64 (100%)
GERIATRIC VISITS		
Yes	48 (80%)	0 (0%)
No	12 (20%)	64 (100%)
GERIATRIC PATIENTS ON PLACEMENT	Intervention (*n* = 50)	Control (*n* = 53)
M (SD)	6.62 (2.67)	6.75 (2.59)
Min - Max	1–16	2–11

### Primary outcome

**Fig. 2:**
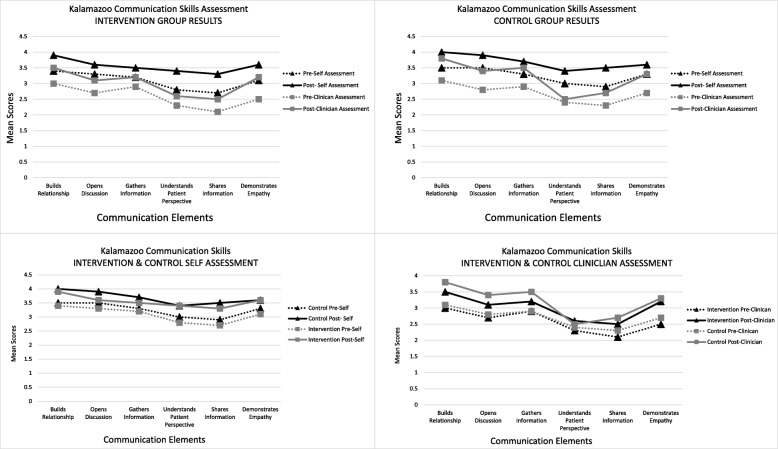
Kalamazoo Communication Skills Assessment Mean Results

The total KCSA mean score for the intervention group clinician rating improved by 2.8 from Time 1, (mean ± SD: 15.4 ± 3.09) to Time 2, (mean ± SD: 18.2 ± 3.20). This was statistically significant (*p* < 0.001), with a large effect size (η^2^ = 0.41). Similarly the control group clinical rating improved by 2.9 from Time 1, (mean ± SD: 16.2 ± 2.01) to Time 2, (mean ± SD: 19.1 ± 3.60). This was also statistically significant (*p* < 0.001), with a large effect size (η^2^ = 0.35). The complete KCSA results are reported in Table 2. An analysis of mean score across all 6 communication domains for both groups found statistically significant improvement and medium to large effects sizes for all raters. A graphical comparison between groups and raters can be found in Fig. 2. Multiple linear regression Model 1 found gender (β = − 0.25; *p* = 0.01) was the best predictor of clinician-assessed communication (KCSA), with females having higher scores. In Model 2 gender in combination with the number of geriatric patients seen on placement (β = − 0.6; *p* = 0.04) was the best predictor of self-assessed communication (KCSA), i.e. the more patients males saw the lower they rated their communication, while still rating themselves much higher than their female counterparts. The regression model results are reported Table 3.

**Table 2:** FAQ2 (Knowledge), ASD (Attitudes) & KCSA (Communication) Results

	INTERVENTION GROUP		CONTROL GROUP		
KCSA TOTAL SCORES^a^					
*Self-Assessment*	M(SD)	Min-Max	M(SD)	Min-Max	p
Time 1	(*n* = 57)		(*n* = 64)		
	18.5 (4.26)	7–30	19.4 (4.64)	9–28	0.26
Time 2	(*n* = 52)		(*n* = 57)		
	21.1 (4.47)	14–30	22.2 (4.45)	13–30	0.19
p	< 0.001		< 0.001		
η^2^	0.31		0.33		
95% Confidence Interval	−3.67 – −1.38		− 3.49 - -1.56		
df	t(50) = −4.79		t(56) = −5.25		
*Clinician-Assessment*	M(SD)	Min-Max	M(SD)	Min-Max	p
Time 1	(*n* = 59)		(*n* = 64)		
	15.4 (3.09)	7–22	16.2 (2.01)	9–22	0.11
Time 2	(*n* = 52)		(*n* = 57)		
	18.2 (3.20)	13–26	19.1 (3.60)	11–25	0.14
p	< 0.001		< 0.001		
η^2^	0.41		0.35		
95% Confidence Interval	−3.96 - -1.96		−3.98 - -1.85		
df	t(51) = −5.96		t(56) = −5.47		
FAQ2^b^	M(SD)	Min-Max	M(SD)	Min-Max	p
	(*n* = 60)		(*n* = 64)		
Time 1	10.1 (1.94)	7–14	9.9 (1.85)	4–14	0.57
	(*n* = 53)		(*n* = 57)		
Time 2	10.5 (2.06)	5–15	9.8 (1.99)	5–14	0.10
p	0.51		0.87		
η^2^	0.01		0.00		
95% Confidence Interval	−0.92 – 0.46		−0.57 – 0.63		
df	t(52) = − 0.66		t(56) = 0.17		
ASD^c^	M(SD)	Min-Max	M(SD)	Min-Max	p
	(*n* = 58)		(*n* = 58)		
Time 1	116.9 (17.09)	74–147	117.7 (15.06)	74–149	0.77
	(*n* = 48)		(*n* = 51)		
Time 2	119.7 (16.45)	82–156	118.4 (16.87)	71–155	0.79
p	0.12		0.58		
η^2^	0.05		0.01		
95% Confidence Interval	−7.00 – 0.83		−4.18 - 2.37		
df	t(47) = −1.58		t(50) = −0.55		

^a^KCSA Total scores can range from 6 (poor) – 30 (excellent)

^b^FAQ2 scored out of 20

^c^ASD neutral attitude 128, lower scores represent more positive attitudes

**Table 3:** Summary of multiple regression analysis predicting KCSA Scores

MODEL 1	KCSA Both Groups Clinician-Assessment F(3, 106) = 3.04; R^2^ = 0.08		
Predictors	Coefficient (95% CI)	Beta	p
GROUP			
Intervention (Reference)	1.00		
Control	1.12 (− 0.15, 2.4)	0.16	0.83
AGE	0.08 (−0.12, 0.27)	0.07	0.46
GENDER			
Female (Reference)	1.00		
Male	−1.74 (−3.06, −0.42)	−0.25	0.01
MODEL 2	KCSA Both Groups Self-Assessment F(3, 87) = 2.4; R^2^ = 0.08		
Predictors		Beta	p
PLACEMENT	−0.01 (−0.46, 0.44)	−0.01	0.95
GENDER			
Female (Reference)	1.00		
Male	4.3 (−0.8, 9.4)	0.5	0.1
PLACEMENT*GENDER	−0.77 (−1.5, − 0.03)	−0.6	0.04

### Secondary outcomes

There was little difference found between the intervention and control groups in FAQ2 scores prior to the intervention. The intervention group scores improved from Time 1, (mean ± SD: 10.1 ± 1.94) to Time 2, (mean ± SD: 10.5 ± 2.06), however this was not statistically significant (*p* = 0.51), with a small effect size (η^2^ = 0.01). The FAQ2 was found to have poor internal consistency with a Cronbach alpha of .38. The full FAQ2 results are reported in Table 2.

Both groups displayed slightly positive attitudes toward older adults at Time 1 prior to the intervention. At Time 2 both groups had a slight decrease in attitudes, while they still remained on the positive side of neutral. Neither change was statistically significant; *p* = 0.12 and *p* = 0.58 respectively. The ASD was found to have excellent internal consistency with a Cronbach alpha of 0.92. The full ASD results are reported in Table 2.

## Discussion

The educational program designed to increase knowledge, attitudes and behaviour toward older people in this study had little discernible impact on the intervention group when compared to the control group. Both groups had negligible change to their already poor knowledge scores and similarly little change to their already slightly positive attitudes. The area with the most notable improvement was in behaviour. Interpersonal communications scores improved by a significant margin, however this was consistent across both groups.

The primary outcome of this study looked at interpersonal communication with older patients. As indicated in Table 2 there were statistically significant improvements in KCSA score for both the intervention and control groups with both self and clinician rated assessments. These results indicate that regardless of the intervention student interpersonal communication skills with older adults improved. While the results do not support an impactful intervention they do suggest that the pre-intervention Time 1 data collection, where students conducted a patient-centred interview with an older adult, could have influenced the results. All students completed this exercise which was an interaction with real independent older adults. In an effort to provide, observe and measure realistic interactions between students and older adults, and a baseline for non-randomised groups, the results were potentially adversely influenced. A 2010 controlled study of 262 UK medical students utilising post measures only found a two-week geriatric clerkship increased observed geriatric assessment Objective Structured Clinical Examinations (OSCE) significantly in comparison to the control group [30]. A post-test only design, while not allowing for pre and post comparison, would have alleviated this issue [31], and will be strongly considered for future studies.

A closer evaluation of the individual communication elements in the KCSA (Fig. 2) indicated relatively uniform improvement across both groups and assessors with the exception of ‘understands the patient’s perspective’. The intervention group showed greater improvements in this element for both the self-assessment (*p* < 0.001) and the clinician assessment (*p* = 0.01). This indicates that part 2 of the intervention; visits with older adults could have influenced the intervention group’s perspective of older people. Understanding a patient’s perspective is said to increases empathy, rapport development and ultimately patient clinical competence and patient satisfaction [12, 32]. This aspect of the intervention would therefore seem to be of value.

Also worthy of discussion are the regression models and the impact of gender on communication. Model 1 indicates that being female is predicative of better communication skills for the clinician assessment (Table 3). This is consistent with other research which describes the communication, empathy and caring skills of females to be generally superior to that of males [32, 33]. Also of interest in Model 2 was the finding that males self-assessed their communication skills much higher than that of female participants (Table 3). This is also consistent with the literature that young males tend to be more confident and overestimate their abilities particularly in communication skills [34]. These factors should therefore be taken into consideration when teaching communication to undergraduate paramedics.

In order to equate these finding to the TBP we need to also look at the secondary outcomes, as knowledge and attitudes are said to influence behaviour. The intervention groups already low mean FAQ2 scores improved by a small margin of 0.4 and the control group scores decreased by 0.1. Neither result was statistically significant nor had a notable effect size. Given the intervention, in particular the GRACC workshop, was designed to improve knowledge about older adult the results were underwhelming. Are these results indicative of the tool itself; the student’s lack of interest in learning important demographic and biopsychosocial factors pertinent to older people, the intervention, or a combination of these factors? The FAQ2 has been historically criticised for poor reliability [35] which was evidenced again in this study with a Cronbach α of 0.38. This has been explained however by the suggestions that such statistical methods may not be appropriate to evaluate internal consistency for instruments such as this with broad areas of content [26]. As for the students, their knowledge development could have been impacted by their propensity to prioritise clinical knowledge and skills over other areas. Other studies using MCQ versions of the FAQ have similarly reported low knowledge scores with minor improvements following educational interventions. A study of 62 US medicine, pharmacy, social work and nursing students reported only a 3% increase in mean scores from 46 to 49% (*p* = 0.04) post a 4-day geriatric care program [36]. Another US study of 100 US nutrition students who participated in 3 structured interviews with older people reported a 6% increase in knowledge score from 50 to 56% (*p* = 0.15) [37]. This is consistent with our findings where students had low baseline knowledge and only a small increase of 2% (51–53%) post the intervention (*p* = 0.51). Much more research is therefore required in this area before any conclusions can be draw about the effectiveness of interventions to improve knowledge about older people.

Previous studies with paramedic students have consistently produced similar baseline results for the ASD with attitudes being slightly positive [8, 38]. A 2016 survey of 871 student paramedics across four universities in Victoria, Australia found that they had only marginally positive attitudes [8]. In this study the attitudes of both the intervention and control groups remaining positive while decreasing by a very small yet non-significant margin. Other studies report similar minor nonsignificant bidirectional variations in attitudes post interventions [39, 40]. The quality of interaction with older adults who are well and independent is suggested to influence attitudes in a positive direction, [41–43] however this is not conclusively supported by our results.

The TPB assertion that increased knowledge and attitudes lead to improved behaviour was not supported in this study. Nor do the results definitively disprove this theory as factors such as the testing instruments, student motivation and participation, placements variations, and study methodology could account for the results. A comparable study of UK medical students, while using different measurements, reported slight improvements in knowledge between groups (*p* = 0.04), decreasing attitudes (*p* = 0.09) and improved geriatric assessment OSCE scores (*p* < 0.001) [30]. The notable difference was that the study conducted the geriatric assessment OSCE’s post the intervention only. The ELT on the other hand was supported by these findings with the communication of all students improving following the patient-centred interview with an older person at Time 1. These results however could also have been impacted by the practice effect, clinical placement experiences and performing clinical scenarios throughout the semester.

Future research needs to focus on the development of educational strategies that not only enhance undergraduate paramedic student interpersonal communication skills but raise their awareness of the importance of these skills to patient care and outcomes. The influence of knowledge and attitudes on behaviour warrants further investigation however this study indicates that experience plays a vital role also and should be explored more thoroughly. Future studies should seek to refine experiential interventions to provide engaging, meaningful and impactful interactions between paramedic students and older people.

### Limitations

The study was limited by inability to fully randomise the groups due to university timetabling constraints. Despite this, we were reassured to see the group demographics and results at baseline were homogeneous. The study was also unable to control for numerous variables outside of the study e.g. the number of older patients seen on placement, the type of cases and individual student’s involvement in each case, attitudes of clinical supervisors, past experience, work experience and variations in experiences with their chosen older adult. Non-blinding of students while unavoidable once the intervention began could also have influence the participants in the intervention groups performance. While asked not to discuss details the intervention group could have discussed what they were doing with participants in the control group thus influencing the results.

## Conclusion

Conducting rigorous controlled educational studies provides numerous complexities and challenges, however despite this we are able to report some important finding which add to what is already know about paramedic students and older patients. This study affirms that paramedic students have poor knowledge and slightly positive attitudes toward older patients. As the first study to observe, measure and report on the interpersonal communication skills of paramedic student’s with ‘real’ older patients we can report that these skills were fair - good at baseline and improved to good - very good post the intervention. All participants in this study conducted a patient centred interview with a real, independently living older person at the Time 1 data collection which resulted in all students improving. Overall improvement was notably better in the ‘understanding the patients perspective element’ for the intervention group who had conducted one-one visits with an older person.

## Abbreviations

ASD: Aging semantic differential
ELT: Experiential learning theory
FAQ2: Facts on aging quiz 2
GRACC: Geriatric respect, awareness, care and compassion workshop
KCSA: Kalamazoo communication skills assessment
MCQ: Multiple choice questions
OSCE: Objective structured clinical examinations
TPB: Theory of planned behaviour
